# The effect of calorie-restriction along with thylakoid membranes of spinach on the gut-brain Axis Pathway and oxidative stress biomarkers in obese women with polycystic ovary syndrome: a Randomized, Double-blind, placebo-controlled clinical trial

**DOI:** 10.1186/s13048-023-01288-x

**Published:** 2023-11-16

**Authors:** Negin Nikrad, Mahdieh Abbasalizad Farhangi, Fatemeh Pourteymour Fard Tabrizi, Maryam Vaezi, Ata Mahmoudpour, Mehran Mesgari-Abbasi

**Affiliations:** 1 Department of Community Nutrition, Faculty of Nutrition, Tabriz University of Medical Sciences, Tabriz, Iran; 2 Drug Applied Research Center, Tabriz University of Medical Sciences, Attar Neyshabouri, Daneshgah Blv, Tabriz, Iran; 3 Nutrition Research Center, Tabriz University of Medical Sciences, Tabriz, Iran; 4 Fellowship Gynecology-Oncology, Women’s Reproductive Health Research Center, Tabriz University of Medical Sciences, Tabriz, Iran; 5 Department of Obstetrics and Gynecology, Alzahra Teaching Hospital, Tabriz University of Medical Sciences, Tabriz, Iran; 6 Department of Anesthesiology, Faculty of Medicine, Tabriz University of Medical Sciences, Tabriz, Iran

**Keywords:** Polycystic ovary syndrome, Thylakoids, Obesity, Spinacia oleracea, Caloric restriction, Brain-gut axis, Blood-brain barrier, Brain-derived neurotrophic factor, Oxidative stress

## Abstract

**Background:**

Women with polycystic ovary syndrome (PCOS) have higher intestinal mucosal permeability, leading to the lipopolysaccharide (LPS) leakage and endotoxemia. This, in turn, leads to oxidative stress (OS) and neuro-inflammation caused by the gut-brain axis, affecting the neurotrophic factors levels such as brain-derived neurotrophic factor (BDNF) and S100 calcium-binding protein B (S100 B) levels. In this study, it was hypothesized that the thylakoid membranes of spinach supplementation along with a hypocaloric diet may have improved the LPS levels, neurotrophic factors, and OS in PCOS patients.

**Methods:**

In this double-blind, randomized, placebo-controlled, and clinical trial, 48 women with obesity and diagnosed with PCOS based on Rotterdam criteria were randomly assigned to thylakoid (N = 21) and placebo groups (N = 23). A personalized hypocaloric diet with 500 calories less than the total energy expenditure was prescribed to all patients. The participants were daily supplemented with either a 5 g/day thylakoid-rich spinach extract or a placebo (5 g cornstarch) for 12 weeks along with a prescribed low-calorie diet. Anthropometric measurements and biochemical parameters were assessed at baseline and after the 12-week intervention.

**Results:**

A statistically significant decrease in the LPS levels (*P* < 0.001) and an increase in the BDNF levels (*P* < 0.001) were recorded for the participants receiving the oral thylakoid supplements and a low-calorie diet. Furthermore, significant decreases were observed in fasting blood glucose, insulin, homeostatic model of assessment for insulin resistance, free testosterone index, and follicle-stimulating hormone / luteinizing hormone ratio in both groups (*P* < 0.05). No significant differences were detected between the two groups regarding the changes in malondialdehyde, catalase, total antioxidant capacity, and S100B levels (*P* > 0.05).

**Conclusions:**

In sum, the thylakoid membranes of spinach supplemented with a hypocaloric diet reduced the LPS levels, increased the BDNF levels, and improved the glycemic profile and sex-hormone levels; however, they had no effects on the OS markers levels after 12 weeks of intervention.

**Supplementary Information:**

The online version contains supplementary material available at 10.1186/s13048-023-01288-x.

## Introduction

Polycystic ovary syndrome (PCOS), which is one of the most commonly diagnosed endocrine disorders, has a prevalence of more than 20% [1]. The diagnosis of PCOS is made by detecting: (1) irregularities in menstrual such as oligomenorrhea or anovulation (primary or secondary amenorrhea), (2) biochemical and/or clinical hyperandrogenism, and (3) polycystic ovarian morphology based on the criteria of Rotterdam when two out of three characters are present with the exclusion of other etiologies [2]. PCOS is a common multifaceted condition stimulated by several reproductive problems (e.g., fecundity [3] and pregnancy complications [4]) that are probably associated with oocyte/embryo [5] and endometrial competence/quality [6]. Other common features such as hyperandrogenemia, insulin resistance (IR), obesity, and oxidative stress (OS) appear to be present in most PCOS patients [7]. IR plays a crucial role in hyperandrogenism development by increasing luteinizing hormone (LH) secretion and reducing sex hormone-binding globulin (SHBG) synthesis in the liver, which leads to the increased testosterone and gonadotropin-releasing hormone (GnRH) levels while reducing the follicle-stimulating hormone (FSH) secretion and preventing the follicle differentiation and endometrial cell development [8].

Women affected by PCOS have higher abundance of the altered gut flora, which could be associated with IR, metabolic disorders, sex hormone disturbances, and particularly dietary habits [9]. Studies on eating habits of women with PCOS have shown that they consume diets high in carbohydrates and fats, especially saturated fat and refined sugars, which is more common in overweight PCOS patients [10]. The given unhealthy dietary patterns can promote the growth of harmful gram-negative bacteria [11]. Gram-negative bacteria secrete lipopolysaccharides (LPS) from their cell walls, which can cause inflammation, IR, obesity, and oxidative stress by leaking into the systemic bloodstream and increasing the reactive oxygen species (ROS) production [12]. OS resulting from ROS can alter the levels of OS biomarkers such as malondialdehyde (MDA), catalase (CAT) levels, and total antioxidant capacity (TAC) which is sensitive to changes in plasma antioxidant levels and IR degree [13].

Gut microbiota and metabolites related to them, on the other hand, cause IR and hyperinsulinemia by triggering the secretion of gut-brain axis peptides [14]. The gut-brain axis facilitates the bidirectional communication between gut microbiota and brain, connecting neurological system and gastrointestinal tract bilaterally [15]. Impaired microbiota dysbiosis can induce neuroinflammation and changes in serum levels of neurotrophins (e.g., brain-derived neurotrophic factor (BDNF) and S100 calcium-binding protein B (S100 B)( [16]. Systemic inflammation and endotoxemia caused by LPS penetration, followed by pro-inflammatory cytokines and immune response, can destroy the tight junction proteins of endothelial cells within the blood–brain barrier (BBB) and promote the BBB permeability [17]. Increased vascular permeability, potentially allowing toxic substances to enter the central nervous system, and resulting in the altered levels brain biomarkers like S100B as an alternative biomarker for BBB integrity impairment and also BDNF [18]. While BDNF levels are regulated by the gut microbiota and BBB permeability, high-fat diets are associated with lower BDNF levels [15]. It has been discovered that BNDF is necessary for the growth of early follicles in the ovary in order to facilitate the nuclear maturation, fertilization, and early embryo growth, and that ovarian BDNF may activate numerous downstream pathways in the oocyte [19].

The thylakoids are the compartments inside the chloroplasts of the green leafy plants including spinach including antioxidant content (e.g., chlorophylls, flavonoids, and carotenoids, and vitamin E) which may help reduce the oxidative stress caused by scavenging ROS [20]. Thylakoid membranes from spinach enhance the secretion of satiety hormones and may affect the reward system by interacting with triglycerides and delaying the absorption of fat [20]. Thylakoids are considered as the physical barriers against the intestinal mucosa – reducing the absorption of macronutrients and methyl-glucose from the intestinal epithelium – and as the relevant factors affecting the appetite and energy metabolism as well as modulating the intestinal microbiota [21]. In a study by Li Y et al. [22], it was found that a chlorophyll-rich spinach extract supplementation reduced the intestinal permeability and LPS leakage and, therefore, it was assumed that chlorophyll successfully reduced the LPS levels and simultaneously decreased the inflammation in the systemic circulation. In another study by Montelius C et al. [21], it was shown that a supplement of thylakoids along with the diet for ten days in the rat experimental model adjusted the intestinal microbiota, improved the condition of the intestinal microbiota, reduced the food intake, and improved the insulin levels.

Taking into account the modulator of gut microbiota and the antioxidant impacts of thylakoids, the observed association of caloric restriction with functional pathways (e.g., lipopolysaccharide biosynthesis), the modulation of gut microbiota disturbance, as well as the reduction of metabolic endotoxemia and the regulation of inflammation [23], it was hypothesized that the administration of thylakoids supplemented with a hypocaloric diet may have synergistically contributed to improving the gut-brain axis and the antioxidant parameters of the women with PCOS. Therefore, this trial mainly aimed to examine the effect of thylakoid intervention on oxidative level, LPS as marker of gut permeability, and BDNF and S100B levels as neurotrophic factors in PCOS women with obesity who were on a low-calorie diet.

## Methods and materials

### Study population

A total of 48 women with obesity and affected by PCOS from the infertility clinic of Al-Zahra hospital and Sheykholrais Polyclinic in Tabriz, Iran were recruited. The patients’ diagnoses were made by a gynecologist based on the Rotterdam criteria [2]. Inclusion criteria were: women aged 20–45 years, with BMI ranged 30–40 kg/m2, and taking oral contraceptive pills. Exclusion criteria were: women experiencing menopausal transition, pregnancy (or having tendency to become pregnant), or lactation, women with an endocrine disorder interfering with the function of the reproductive system (e.g., diabetes, thyroid, liver, adrenocortical dysfunction, hyperprolactinemia, Cushing’s syndrome, or adrenal hyperplasia), those who were smokers or passive smokers, women using ovulation induction agents or taking blood pressure regulating drugs, and those receiving fertility therapy or willing to receive it. In addition, women taking insulin-sensitizing medications, insulin infusion drugs, vitamin and mineral supplements, antioxidants, or any supplements affecting their body weight, as well as those following certain diets during two months prior to study were excluded from the present study. All participants in the present study, as mentioned earlier, were on oral contraceptive pills prescribed by a gynecologist for their medical treatment, and they were asked to keep their regular medication during the intervention period of the present study. The sample size was calculated using the following formula: $\text{N}=\frac{{\left( {\text{Z}}_{\left(1-\frac{{\upalpha }}{2}\right)}+ {\text{Z}}_{\left(1-{\upbeta }\right)}\right)}^{2}({\text{S}}_{1}^{2}+{\text{S}}_{2}^{2})}{{({{\upmu }}_{1}-{{\upmu }}_{2})}^{2}}$ where N, number of individuals, Z, the value from the table of probabilities of the standard normal distribution for the desired confidence level; β, power of the test; α, statistical significance level; S, standard deviation and µ, variable mean.

This formula was used by taking into account the results of mean ± SD for MDA and the previous result of oxidative stress in PCOS [24] as well as by assuming an α error of 0.05 and the power of 80%. Thus, the total sample size was determined to be 44, which was increased to 48 by predicting a 10% dropout rate.

### Spinach extract preparation

Thylakoid membranes were extracted from the fresh baby spinach (i.e., Spinacia oleracea) leaves. The leaves were then homogenized, filtered, and diluted with distilled water for ten times. The pH was adjusted to 4.7, which allowed the maximum precipitation. The green precipitate was collected, purified, washed using repeated centrifugation technique, and dried. The green thylakoid powder was manufactured by Darook Pharmaceutical Co., Tehran, Iran. Placebo sachets containing corn starch were colored with edible green color and flavored with kiwi fruit flavor (similar to the flavor added to thylakoid powder) so that the two powders could not be distinguishable in terms of appearance and taste. The packages containing the sachets were coded and distributed monthly by a third party who had no other role in performing the study. The participants’ adherence to taking the sachets on a monthly basis was assessed by counting the sachets returned by them. Consuming more than 80% of the sachets was considered as an acceptable adherence effort.

### Study design

The current study was a double-blind, randomized, placebo-controlled, and clinical trial. The present randomized controlled trial was conducted in accordance with the Consolidated Standards of Reporting Trials (CONSORT) 2010 statement (Supplementary Material) [25]. The study protocol was registered on the Iranian Registry of Clinical Trials system under code number IRCT20140907019082N9, and was also approved by the ethics committee of the research vice-chancellor of Tabriz University of Medical Sciences, Tabriz, Iran with a code of ethics (IR.TBZMED.REC.1401.088). A written, informed consent was obtained from all study participants. Eligible women were randomly assigned to two treatment conditions in a ratio of 1:1 with varying block sizes stratified by age and BMI adopting the block randomization method, and sequential allocation was performed using the random allocation software (RAS). Sequentially numbered opaque sealed envelopes are used to perform allocation concealment. The envelopes are opened sequentially in the order of entrance at the baseline of the study and the grouping of participants is decided by the envelope carrying the allocation plan inside it. Until the final analysis, the researchers and patients were kept concealed regarding the randomization and distribution. For both control and thylakoid groups, a hypocaloric diet with 500 calories less than the total energy expenditure (TEE) was designed by a nutrition consultant taking into account the individual characteristics of the participants and using the Mifflin formula. All participants were provided with thylakoid-rich spinach extract or raw corn starch as placebo in sachets, and were instructed to dissolve one sachet in a glass of water and consume it on a daily basis 30 min before the lunch for 12 weeks while following the prescribed low-calorie diet.

### Outcomes

The primary study outcome was to measure and compare the value LPS, neurotrophic factors such as BDNF and S100B levels and also oxidative stress markers like MDA, TAC and CAT among PCOS individuals at the baseline and end of the intervention period of thylakoid supplementation along with low-calorie diet in two groups of intervention and placebo. Secondary outcome measures were evaluation and comparison of glycemic markers, including FBG, insulin and HOMA-IR and hormonal profiles, including the FSH/LH ratio, FTI and DHEA-S in two groups of the study at the baseline and after 12 weeks of intervention.

### Anthropometric, body composition and physical activity measurements

The height, weight, waist and hip circumference and body composition of the participants were measured at the beginning, sixth week after the study, and at the end of the study by a trained person to lessen the individual error. BMI was calculated as the body weight (kg) divided by the square of the height (m). Body composition was measured performing bioelectrical impedance analysis and using Tanita MC-780 SMA (Tanita Corporation, Tokyo, Japan). The International Physical Activity Questionnaire-Short Form (IPAQ-SF) was used to measure the level of physical activity at baseline, 6th week, and end of the intervention. This validated questionnaire records self-reported activity at four levels of intensity, namely high-intensity activity (e.g., aerobics), moderate-intensity activity (e.g., leisure cycling), walking, and sitting [26].

### Biochemical analyses

At the baseline and end of the study, a 10 mL venous blood sample was taken from all participants after 12 h of fasting. Blood sampling was performed between 8:00 AM and 9:00 AM during the early follicular phase (days 2 to 5) of a spontaneous menstrual cycle or P-induced menstrual cycle. Blood samples were immediately centrifuged at a speed of 3500 rpm for ten minutes, and serum samples were separated from whole blood and directly frozen at -80 °C until examination time. Oxidant and antioxidant parameters, including CAT (Teb Pazhouhan Razi Co., Catalog Number: TPR-CAT-96T, Tehran, Iran. Intra and inter-assay coefficient of variation (CV) 4.1% and 9.9% respectively), TAC (Teb Pazhouhan Razi Co., Catalog Number: TPR-TAC-96T, Tehran, Iran. Intra and inter-assay CVs 5.7% and 3.7% respectively), and MDA (Teb Pazhouhan Razi Co., Catalog Number: TPR-MDA-96T, Tehran, Iran. Intra and inter-assay CVs 6.7% and 7.2% respectively), were measured using enzyme-linked immunosorbent assay (ELISA) kit and based on the manufacturer’s instructions. The serum fasting blood sugar (FBS) was measured adopting enzymatic methods and colorimetric technique and using commercial kits (Pars Azmoon, Catalog Number:1,500,017, Karaj, Iran. Intra- and inter-assay CVs were 1.63 and 2.2 respectively) with an auto-analyzer (Hitachi-917, Tokyo, Japan). Furthermore, serum insulin level (Monobind, Inc.,Catalog Number: 2,425,300, Lake Forest, CA, USA. Intra and inter-assay CVs < 5.6% both) was determined by performing chemiluminescence (IMMULITE 2000, SIEMENS), and the homeostatic model of assessment for insulin resistance (HOMA-IR) was calculated using the suggested formula [27]. Hormonal profiles, including the follicle stimulating hormone, luteinizing hormone, total testosterone, dehydroepiandrosterone sulfate, and sex hormone binding globulin, were measured using ELISA kits (Bioassay Technology Laboratory, Shanghai Korean Biotech, Shanghai City, China. Catalog Numbers: E1001Hu, E1037Hu, E1036H, E1057Hu, E1011Hu respectively, Intra and inter-assay CVs < 5.0% for these measurements). Free testosterone index (FTI) was calculated using the formula: FTI = 100 x serum testosterone (nmol/L)/sex hormone binding globulin (SHBG, nmol/L). The serum BDNF (Padginteb Co., Catalog Number: CSB-E04501h, Iran. Intra-assay CV < 8% and CV < 10%), S100B (Biovendor, Catalog Number: RD192090100R, Heidelberg, Germany. Intra- and inter-assay CVs were 2.0 and 5.9 respectively.) and LPS (Bioassay Technology Laboratory, Shanghai Korean Biotech. Catalog Number: E1791H, Shanghai City, China. The intra and inter-assay CVs were 5.6% and 6.9% respectively) levels were determined by adopting sandwich ELISA method and following the manufacturer’s recommendation for the commercial kit.

### Statistical analysis

Data in the text and tables were presented as means ± standard deviation and were analyzed using SPSS version 23 (SPSS Inc., Chicago, IL). After testing the normal distribution using the Kolmogorov–Smirnov test, independent sample t-tests or Mann–Whitney or chi squared tests were performed to determine the between-group differences at baseline. The analysis of covariance (ANCOVA) was performed to compare the two groups after 12 weeks of intervention after adjusting for baseline values of each variable and potential confounders such as age, changes in weight and body mass index during 12 weeks, and physical activity as well as reporting the crude *P*-value at the end of the trial. Meanwhile, one-way ANCOVA was performed to estimate the effect size by taking the difference in the means of two study groups and dividing by the pooled standard deviation. Independent t-test was used for reporting mean difference (MD) with a 95% confidence interval (CI) for between-group differences. *P*-values less than 0.05 were considered statistically significant.

## Results

### Participant’s characteristics

**Fig. 1:**
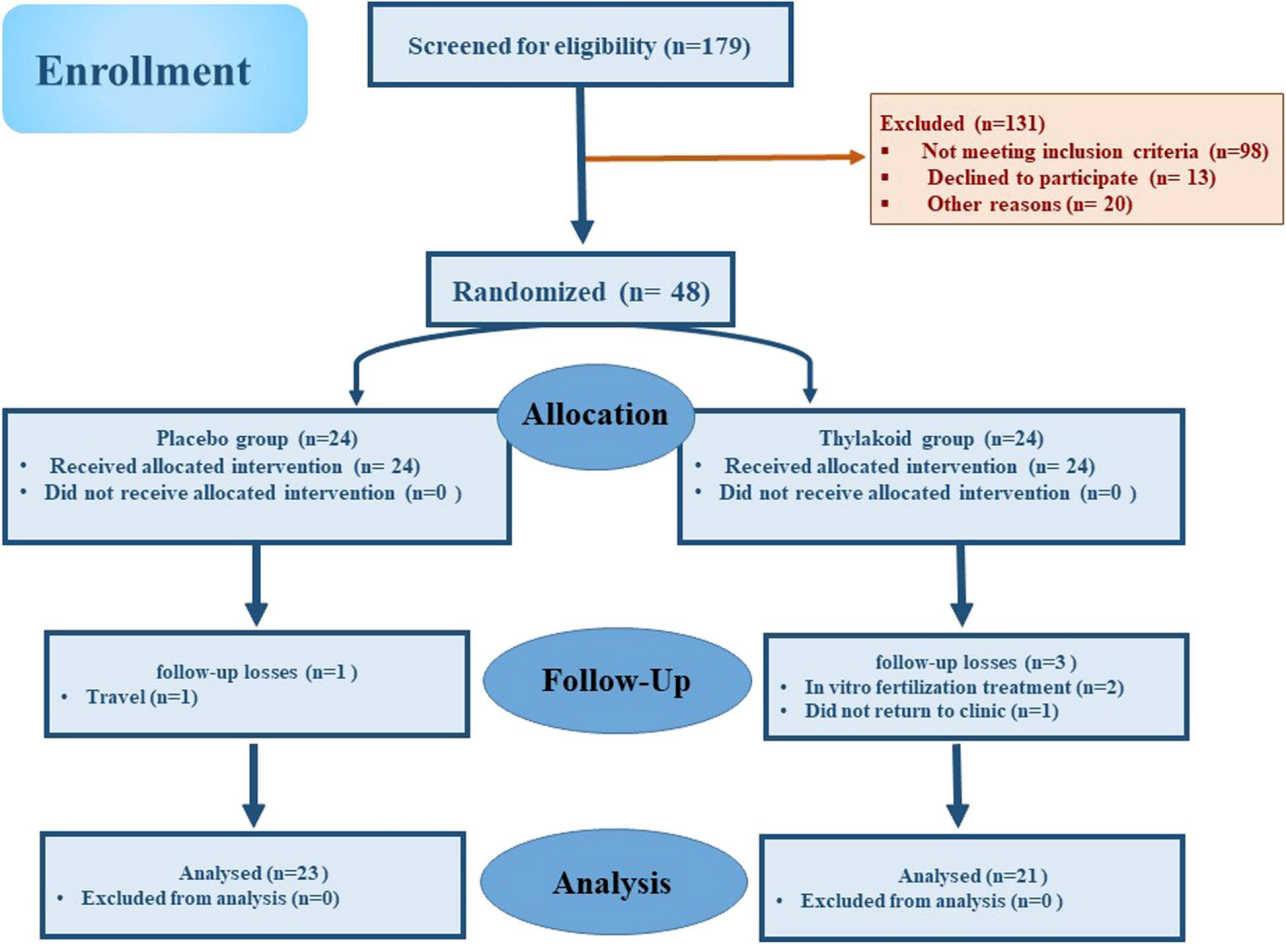
Study Flow Diagram

A total of 179 individuals were screened, out of who 48 eligible ones were randomized into two groups, namely the placebo group (n = 24) and the thylakoid group (n = 24); however, three subjects from thylakoid group (due to following in vitro fertilization treatment and failure to follow up) and one subject from placebo group (due to travelling) were dropped out of the study. Therefore, the data of 44 participants were analyzed and reported [thylakoid (n = 21) and placebo (n = 23)] (Fig. 1). To assess the participants’ compliance, they were asked to return the unused sachets. The sachet counts revealed the high compliance, since the participants in both groups had consumed more than 90% of the sachets distributed during the trial. During 12 weeks of supplementation with thylakoid or placebo, no negative side effects or symptoms were observed, hence there was no need for an interim analysis or terminating instructions. As shown in Table 1, no significant difference was detected between the two groups in terms of the baseline characteristics of participants including age, weight, and height. Furthermore, no difference was observed between the thylakoid and placebo groups regarding the duration of PCOS age at menarche and blood pressure at the beginning of the study (Table 1, P ≥ 0.05).

**Table 1:** Baseline characteristics of participants and comparison of anthropometric indices and dietary intake of the study population at the baseline

Variables	Thylakoid group (n = 21)	Placebo group (n = 23)	P*
**Age (y)**	31.86(2.35)	32.04 (2.83)	0.812
**PCOS duration (years)**	12 (2.05)	11.52 (1.59	0.391
**Age at menarche (y)**	12.40 (0.46)	12.33 (0.45)	0.595
**Blood pressure (mmHg)**			
**Systolic**	125.95 (6.64)	125.47 (6.79)	0.820
**Diastolic**	92.01 (3.66)	91.81 (3.07)	0.842
**Weight (kg)**	89.21 (6.50)	88.14 (7.27)	0.613
**Height (cm)**	159.33 (3.52)	158.00 (3.43)	0.216
**BMI (kg/m2)**	35.13 (2.16)	35.31 (2.77)	0.808
**WC (cm)**	108.09 (3.89)	108.18 (4.22)	0.944
**WHR**	0.92 (0.01)	0.93 (0.01)	**0.022**
**FM (kg)**	32.40 (3.46)	32.17 (3.90)	0.843
**FFM (kg)**	56.79 (3.21)	55.96 (3.53)	0.422
**Energy (Kcal)**	2346.3 (399.96)	2297.8 (446.26)	0.707
**Carbohydrates (g)**	333.24 (57.00)	326.82 (62.21)	0.724
**Protein (g)**	63.72 (13.93)	62.48 (15.56)	0.784
**Fat (g)**	32.37 (1.30)	32.24 (1.58)	0.662
**Fiber (g)**	19.16 (5.52)	20.96 (3.88)	0.213
**Cholesterol (mg)**	202.70 (39.18)	198.20 (44.45)	0.725

BMI, Body mass index; WC, Waist Circumference; WHR, waist-to-hip ratio; FM, Fat Mass; FFM, Fat Free Mass. All values are presented as mean (SD). *P based on independent sample t-test

### Anthropometric parameters and dietary intakes

**Fig. 2:**
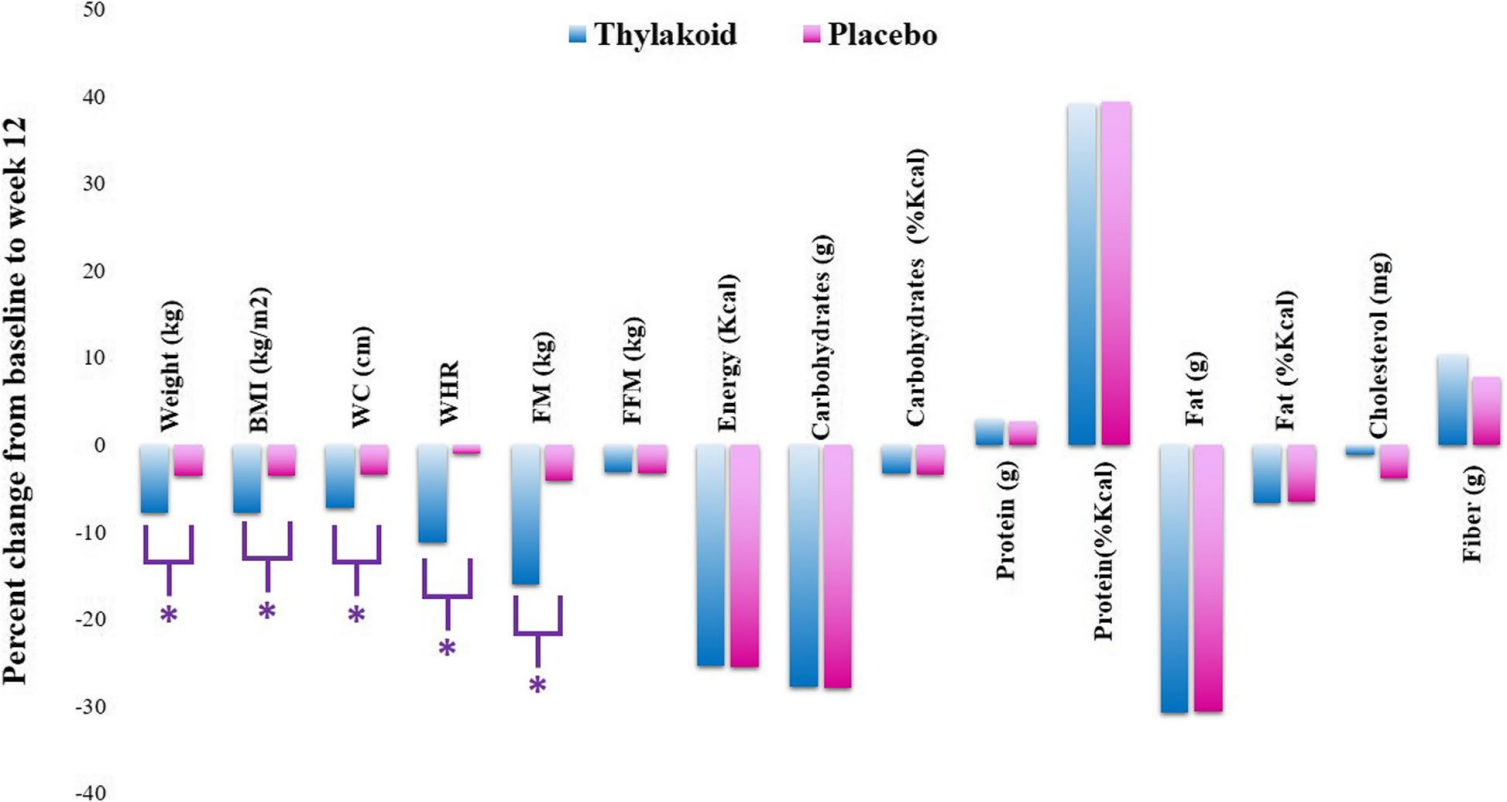
Percent Changes in Dietary Intakes after 12 Weeks of Intervention BMI: Body mass index; WC: Waist Circumference; WHR: waist-to-hip ratio; FM: Fat Mass; FFM: Fat Free Mass. *Significantly higher vs. baseline obtained from intra-group-paired analyses, the paired t-test (*P* ≤ 0.05)

According to our results, there was no significant difference between the two groups in terms of the anthropometric measurements including weight, body mass index, fat mass, fat-free mass, and WC at baseline (*P* > 0.05) except for WHR (*P* = 0.022). The percent change in anthropometric indices as well as the dietary intake of study population in thylakoid membranes of spinach and placebo groups at the end of the study are demonstrated in Fig. 2. At the beginning of the study, no significant difference was detected between the two groups regarding the mean intake of energy, macronutrient and fiber, and cholesterol (*P* ≥ 0.05). After a 12-week intervention and implementing a caloric restriction on both intervention and control groups, body weight decreased from 89.21 ± 6.50 kg to 82.23 ± 6.16 kg in thylakoid group, and from 88.14 ± 7.27 kg to 84.95 ± 6.87 kg in placebo group (*P* < 0.001). Similar to body weight, other anthropometric indices such as BMI, WC, WHR, and FM were reduced in both groups (Fig. 2). According to results of the within-group analyses, the energy intake in response to the low-calorie diet showed a significant reduction in both groups after 12 weeks compared to baseline. While the calorie intake in the intervention group was decreased from 2346.3 ± 339.96 kcal to 1749.37 ± 117.60 kcal (*P* < 0.001), this intake in the placebo group was decreased from 2297.8 ± 446.8 kcal to 1711.3 ± 119.84 kcal (*P* < 0.001). Meanwhile, a significant decrease was found in the macronutrient intake, except for protein in response to the 12-week intervention (Fig. 2).

### Glycemic markers and hormonal parameters

Examination of the glycemic markers and sex hormones [28] revealed a significant reduction in FBG, insulin, HOMA-IR, FTI, and FSH/LH levels in both thylakoid and placebo groups after adjustment for potential confounding factors (*P* > 0.05). However, there were no significant differences between DHEA-S after 12 weeks of intervention and baseline in the placebo group (*P* = 0.730), after adjustment for baseline level of DHEA-S, age, changes in weight and BMI during 12 weeks, and physical activity the differences remain non-significant (*P* = 0.996). Interestingly, the DHEA-S levels decreased after thylakoid supplementation (*P* = 0.040); however, the decreasing trend was halted after the adjustment for the given confounding factors (*P* = 0.104) (Table 2). The difference between thylakoid and placebo groups in terms of the baseline values of biochemical variables was not statistically significant (P ≥ 0.05).

**Table 2:** Comparison of biochemical parameters between and within the thylakoid and the placebo group at baseline and after the intervention

Variable	Normal ranges	Period	Thylakoid group (n = 21)	Placebo group (n = 23)	MD (95% CI)	P value	Effect size
**FBG** **(mg/dl)**	70–99	**Baseline**	93.09 (5.39)	96.56 (7.43)	-3.47 (-7.46, 0.52)	0.13	0.05
		**End**	92.66 (4.77)	96.08 (6.09)	-3.42 (-6.75, -0.08)	0.13	
		**MD (95% CI)**	-0.43 (-1.05, 0.19)	-0.48 (-1.36, 0.40)			
		**P value**	0.165^*^ **< 0.001** ^**^	0.273^*^ **< 0.001** ^**^			
**Insulin** **(µU/mL)**	5–15	**Baseline**	17.97 (2.45)	18.63 (2.33)	-0.65 (-2.11, 0.80)	0.52	0.74
		**End**	12.57 (2.11)	17.43 (2.25)	-4.86 (-6.19, -3.52)	0.88	
		**MD (95% CI)**	-5.40 (-6.25, -4.55)	-1.19 (-1.56, -0.83)			
		**P value**	**< 0.001** ^*^ **0.006** ^**^	**< 0.001** ^*^ **< 0.001** ^**^			
**HOMA-IR**	< 1	**Baseline**	4.14 (0.69)	4.43 (0.59)	-0.29 (-0.68, 0.10)	0.54	0.75
		**End**	2.87 (0.49)	4.12 (0.53)	-1.25 (-1.56, -0.93)	0.72	
		**MD (95% CI)**	-1.27 (-1.48, -1.05)	-0.31 (-0.42, -0.19)			
		**P value**	**< 0.001** ^*^ 0.003**	**< 0.001** ^*^ **< 0.001** ^**^			
**DHEA-S** **(µg/dl)**	45–270	**Baseline**	246.09 (9.58)	242.56 (7.42)	3.53 (-1.66, 8.72)	0.18	**< 0.01**
		**End**	241.66 (5.65)	241.91 (5.88)	-0.24 (-3.76, 3.27)	0.73	
		**MD (95% CI)**	-4.43 (-8.64, -0.22)	-0.65 (-4.52, 3.21)			
		**P value**	**0.040** ^*^ 0.104^**^	0.730^*^ 0.996^**^			
**FTI**	0.34–12.36	**Baseline**	8.82 (0.97)	9.28 (1.07)	-0.46 (-1.08, 0.16)	0.59	0.47
		**End**	7.86 (0.48)	8.83 (0.93)	-0.96 (-1.42, -0.50)	**< 0.01**	
		**MD (95% CI)**	-0.96 (-1.24, -0.66)	-0.45 (-0.58, -0.31)			
		**P value**	**< 0.001** ^*^ **0.001** ^**^	**< 0.001** ^*^ **< 0.001** ^**^			
**FSH/LH**	1–2	**Baseline**	0.57 (0.05)	0.58 (0.05)	-0.01 (-0.06, 0.01)	0.06	**< 0.01**
		**End**	0.56 (0.05)	0.56 (0.05)	0.00 (-0.06, 0.01)	0.07	
		**MD (95% CI)**	-0.01 (-0.00, 0.00)	-0.02 (-0.00, 0.00)			
		**P value**	0.582^*^ **< 0.001** ^**^	0.578^*^ **< 0.001** ^**^			
**BDNF** **(ng/ml)**	8–46	**Baseline**	14.05 (1.25)	13.63 (1.10)	0.42 (-0.31, 1.16)	0.41	0.26
		**End**	15.34 (1.73)	13.48 (1.17)	1.85 (0.90, 2.79)	0.34	
		**MD (95% CI)**	1.29 (0.47, 1.95)	-0.14 (-0.97, 0.69)			
		**P value**	**0.003** ^*^ **< 0.001** ^**^	0.728^*^ 0.084^**^			
**LPS** **(EU/ml)**	0.15–0.35	**Baseline**	2.35 (0.20)	2.35 (0.22)	0.01 (-0.82, 0.9)	0.25	0.38
		**End**	2.21 (0.23)	2.55 (0.23)	-0.34 (-1.90, 1.12)	0.40	
		**MD (95% CI)**	-0.14 (-0.27, -0.02)	0.21 (-1.46, 1.87)			
		**P value**	0.022* **< 0.001** ^**^	**0.012** ^*^ 0.174^**^			
**S100B** **(ng/ml)**	0.02–0.15	**Baseline**	0.14 (0.02)	0.13 (0.04)	0.01 (-0.12, 0.36)	0.05	**< 0.01**
		**End**	0.13 (0.04)	0.13 (0.04)	0.01 (-0.32, 0.36)	**0.02**	
		**MD (95% CI)**	-0.01 (-0.01, 0.02)	-0.00 (-0.03, 0.03)			
		**P value**	0.377^*^ 0.349^**^	0.916^*^ 0.536^**^			
**Catalase** **(nmol/min/ml)**	2–35	**Baseline**	13.64 (8.04)	13.95 (9.08)	-0.31 (-5.25, 5.61)	0.57	0.09
		**End**	15.98 (9.06)	10.80 (7.78)	5.18 (-1.80, 9.12)	0.45	
		**MD (95% CI)**	2.34 (-4.15, 8.84)	-3.15 (-8.03, 1.72)			
		**P value**	0.458* 0.595**	0.193^*^ 0.081^**^			
**TAC** **(mmol/l)**	0.3–0.46	**Baseline**	1.40 (0.52)	1.53 (0.70)	-0.13 (-0.51, 0.24)	0.05	**0.04**
		**End**	1.44 (0.58)	1.70 (0.54)	-0.26 (-0.59, 0.08)	0.39	
		**MD (95% CI)**	0.04 (-0.26, 0.34)	0.16 (0.16, 0.49)			
		**P value**	0.773* 0.519**	0.303^*^ 0.880^**^			
**MDA** **(nmol/ml)**	6.2–26.0	**Baseline**	5.12 (1.76)	4.18 (1.34)	0.93 (-2.10, 3.98)	0.79	**0.02**
		**End**	3.57 (1.58)	2.77 (1.95)	0.80 (-0.95, 3.22)	0.29	
		**MD (95% CI)**	-1.55 (-6.42, 2.54)	-1.41 (-3.83, -0.98)			
		**P value**	0.331^*^ 0.446^**^	0.198^*^ 0.067^**^			

FBG, fasting blood glucose; HOMA-IR, homeostatic model assessment for insulin resistance; DHEAS, dehydroepiandrosterone sulfate; FTI, free testosterone index; LH, luteinizing hormone; FSH, follicle stimulating hormone; BDNF, brain-derived neurotrophic factor; LPS, lipopolysaccharides; S100B; S100 calcium-binding protein B; CAT, catalase; TAC, total antioxidant capacity; MDA; malondialdehyde; SD, standard deviation; MD, mean difference, CI, confidence interval. P* values derived from unadjusted ANCOVA P** values derived from ANCOVA after adjustment for confounders (baseline values, age, changes in weight and body mass index during 12 weeks, and physical activity)

### Biochemical parameters

As for the biochemical parameters, the brain-derived neurotrophic factor levels in the thylakoid group significantly increased from 14.05 ± 1.25 ng/mL to 15.34 ± 1.73 ng/mL (*P* = 0.003) compared to those in the placebo group (*P* < 0.001). This significant increase was continued after adjustment for covariates including baseline BDNF serum level, age, changes in weight and body mass index during 12 weeks, and physical activity (*P* < 0.001). Remarkably, serum LPS of the thylakoid group significantly decreased at week 12 (22.19 ± 2.32 EU/mL) compared to the baseline levels (23.57 ± 2.07 EU/mL) (unadjusted *P* = 0.022 and adjusted *P* < 0.001); however, LPS levels in the placebo group increased from 23.51 ± 2.27 EU/mL to 25.58 ± 2.39 EU/mL (*P* = 0.012), and this increasing trend was halted after the adjustment of confounding variables. Moreover, no significant difference was observed in the levels of catalase, total antioxidant capacity, and malondialdehyde in both groups after 12 weeks of intervention compared to baseline even after the adjustment for confounders (*P* > 0.05). Although the S100B serum level in the supplement group decreased from 0.14 to 0.13, the difference was not statistically significant based on the between-group analysis adjusted for the baseline value of S100B and confounders. A low estimated effect size of intervention on the DHEA-S, FSH/LH, S100B, TAC, MDA (η²< 0.05) was observed in Table 2. In this study, no harm was recorded for the participants with PCOS consuming 5 g of thylakoids on a daily basis before the lunch for 12 weeks.

## Discussion

This study mainly aimed to examine the effect of thylakoid supplementation combined with a low-calorie diet on the gut-brain axis by evaluating the levels of neurotrophic factors such as the brain-derived neurotrophic factor (BDNF) and S100 calcium-binding protein B (S100 B) in order to assess neuroinflammation caused by LPS secreted from gut microbiota in PCOS women with obesity. This study also aimed to investigate the oxidative stress markers such as malondialdehyde (MDA), catalase (CAT) levels and total antioxidant capacity (TAC) after 12 weeks of intervention. The consumption of low-calorie diet with 5 g/day thylakoid membranes of spinach during 12 weeks significantly increased the BDNF levels and decreased the LPS levels, suggesting a regulatory effect of this intervention on the gut-brain axis. Furthermore, the intervention carried out in this study had beneficial effects on the glycemic markers and sex hormones. However, the thylakoid membranes of spinach with a hypo-caloric diet had no significant effect on the oxidative stress markers in PCOS patients with obesity, which rejected our hypotheses.

The observed reduction in the LPS levels in this trial may have been attributed to the capability of thylakoids to reduce the fat absorption and delay the digestion of fat [29], given that dietary fat increases LPS absorption [30], thus, the observed decrease in the LPS levels may have been due to the role of thylakoid supplementation in reducing the fat absorption. Montelius C et al. [31] also demonstrated that the thylakoids membranes may have proved useful in controlling the intestinal absorption, suggesting the barrier function of the intestine, and preventing the leakage of LPS into systemic circulation, which is supported by the finding indicating that the chloroplast thylakoids membranes reduce the macromolecular permeability. This effect was later discovered to occur predominantly in the diet-induced obese mice, as demonstrated by Li Y et al. [22]. Supplementation of a chlorophyll-rich spinach extract may decrease the gut permeability and LPS leakage. Since LPS can induce an oxidative stress in the cells after being absorbed into the systemic bloodstream, LPS binding protein (LBP) binds to the CD14 toll-like receptor complex (TRL-4) on the innate immune cells’ surfaces, activating the signaling pathway downstream and resulting in immune system activation [14]. Reactive oxygen species (ROS) are believed to be involved in the mechanism of the LPS toxic effects, resulting from a significant decrease in antioxidant defense enzymes such as catalase (CAT), which converts ROS to less harmful molecules [32]. According to a study from Iran, patients with PCOS experience elevated oxidative stress as opposed to the healthy controls of the same age and BMI, and individuals with PCOS have significantly greater levels of advanced oxidation protein products and significantly lower levels of total antioxidant status [33]. However, it is not yet certain if the reduction of endotoxemia caused by the reduction of LPS leakage can improve the oxidative status of PCOS patients. It could be an indicator of the antioxidant enzyme inhibition in PCOS patients with IR, which may be associated with the presence of an advanced-stage PCOS and prevent the changes in the antioxidant enzyme concentrations [34]. Palomba S. [3] argues that the PCOS women have an increased OS and elevated production of ROS that increase the incidence of meiotic abnormalities, altered final oocyte quality. Therefore, it is recommended that strategies improving the oocyte maturation and quality should be adopted in treating women with PCOS. As expected, the BDNF levels were significantly increased in thylakoid group compared to placebo group. Low concentrations of BDNF in the ovarian follicle of the PCOS rats may be the underlying cause of the follicular development abnormalities, which implies that BDNF may be a physiological modulator stimulating follicle development, granulosa cell proliferation, and oocyte maturation [19].

It has been also confirmed that the increase of inflammatory cytokines as a result of TRL-4 activation by LPS reduces the BDNF mRNA expression [35]. These findings raise the possibility that significant beneficial changes in the intestinal permeability to LPS and its leakage into the circulation prevent the systemic inflammation and OS, and increase the brain neurotrophic factor levels in PCOS patients. Zhang J et al. [36] reported an imbalance in the intestinal microbiota, with significant high levels of several gram-negative bacteria in the gut of the PCOS women with obesity. Tremellen et al. [11] proposed the “DOGMA” (dysbiosis of gut microbiota) hypothesis, which describes the following probable progression of events in the pathophysiology of PCOS: Obesity or a diet high in sugar and fat, as well as a low fiber diet disrupts intestinal flora, disrupts the junction between intestinal epithelial cells (Loosen the tight junction) and increasing the permeability of the intestinal mucosa. Leaky intestinal could promote LPS leakage into blood circulation, and results in immune system response that impairs with insulin receptor function, resulting in IR, and finally IR/Hyperinsulinemia may stimulate testosterone release, interfering with follicular growth.

According to our study results, decreased free testosterone index (FTI), FSH/LH ratio, and DHEA-S were also observed in both placebo and thylakoid groups, which may have improved hyperandrogenism and increased the number of mature oocytes. As Insenser M et al. [37] argue, the sex hormone levels are associated with the changes in the gut microbiome, and the gut microbiota may contribute to the regulation of sex hormones.

**Fig. 3:**
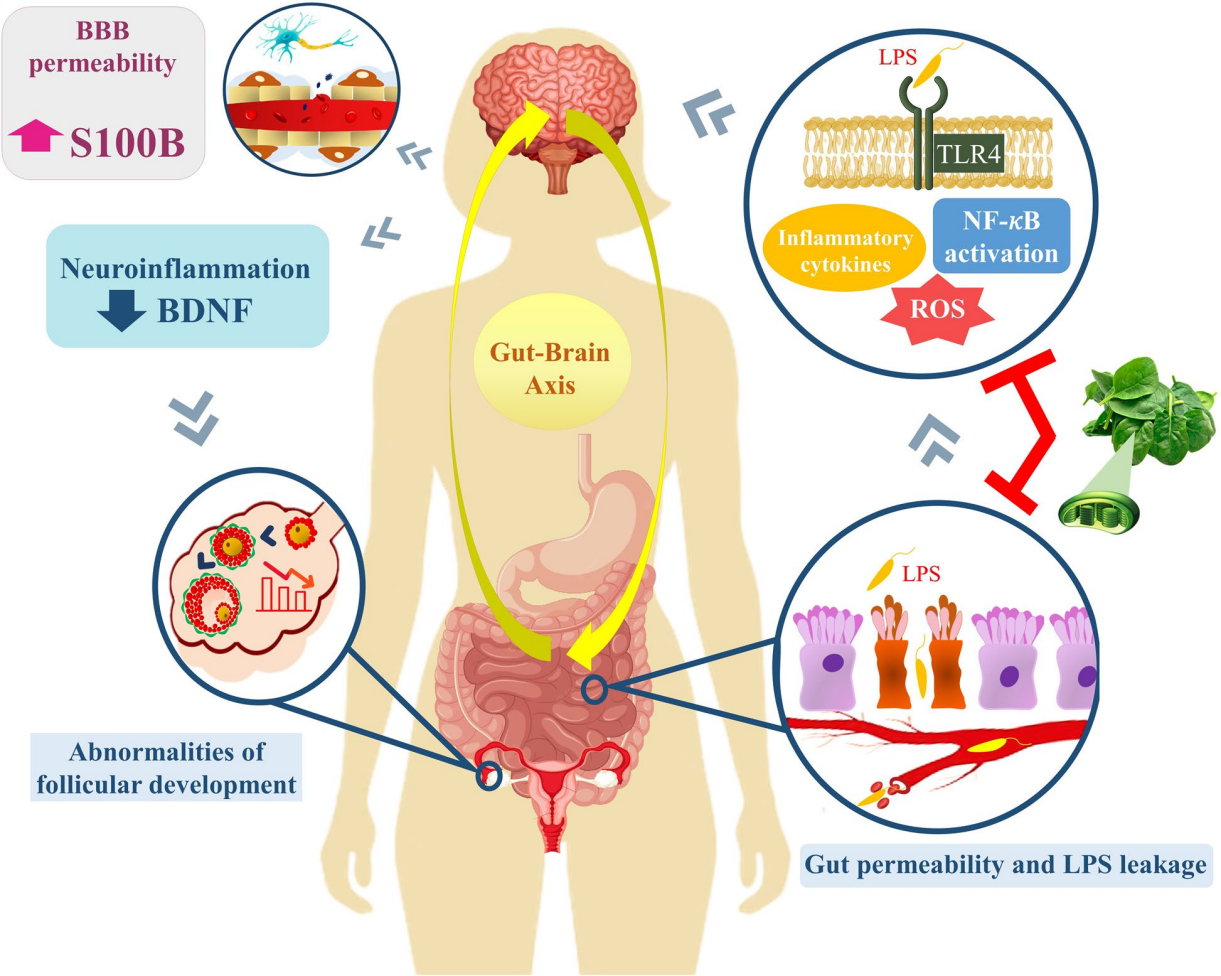
Intestinal permeability led to leakage of LPS intothe systemic circulation and stimulated the immune response and neuro-inflammation, which further increase the BBB permeability and deregulation in serum levels of S100B; also neuro-inflammation was able to alter the BDNF levels, which may have caused disorders in follicular growth. By interfering with LPS leakage and antioxidant properties, thylakoids may have impaired this series of disorders in PCOS patients Abbreviations: BBB: blood-brain barrier; BDNF: brain-derived neurotrophic factor; LPS: lipopolysaccharides; NF-κB: nuclear factor kappa B; ROS: reactive oxygen species; S100B: S100 calcium-binding protein B

To our knowledge, in this regard, no previous human studies evaluated the effects of thylakoid membranes spinach supplementation combined with hypo-calorie diet on oxidative stress and biomarkers of gut-brain axis in individuals with PCOS. The following studies are those studies investigating the thylakoid effects: Montelius C et al. revealed that consuming 5 g of thylakoid per day resulted in the body weight reduction and reduced the desire for sweets and chocolate in individuals with overweight or obesity [38]. In a 12-week double-blind intervention consisting of 5 g/day of the thylakoid membranes from spinach powder with low-calorie diet, Tabrizi FP et al. documented significant decreases in weight, waist circumference, fat mass, and insulin levels in PCOS individuals from the spinach-derived thylakoid group compared to the placebo group [28]. Some of the mechanistic pathways of the possible beneficial effects of thylakoid are presented in Fig. 3.

The strength of our study lies in the fact that its samples were selected from the multi-ethnic obstetrics and gynecology centers with clients from the suburbs in order to increase the generalizability of its results. Furthermore, the biweekly follow-ups performed by a dietitian as well as the examination of the patients’ problems during the intervention increased the patients’ cooperation.

Our study faced few limitations. The main limitation of our study was its relatively small sample size; therefore, it was recommended that this study should be considered as a pilot study with encouraging results, and that further trials with larger sample sizes should be conducted to confirm or disconfirm our results. Second, the biochemical parameters were determined at a single time point in our study, whereas the repeated and longer measurements may have provided a stronger determination of the physiological mechanisms affecting the gut-brain axis. Third, the loads of fecal bacteria and microbiome characterization before, during, and after intervention were not determined in this study to investigate the composition of intestinal microflora. Fourth, only two markers of oxidative stress CAT, TAC and MDA were evaluated in our study; therefore, it was recommended that future studies should be carried out to investigate the remaining parameters (e.g., superoxide dismutase (SOD) and C-reactive protein (CRP)). It should be also noted that it was difficult to ensure the individuals’ adherence to a calorie-restricted diet for 12 weeks in this study.

## Conclusion

It was concluded that a prescribed hypo-calorie diet supplemented with 5 gr thylakoid membranes of spinach significantly decreased the serum LPS levels, increased the BDNF levels, improved the fasting blood glucose and insulin sensitivity, and exerted beneficial effects on the sex-hormonal status in obese individuals with PCOS. This intervention, however, failed to document significant changes in S100B and oxidative stress markers such as CAT, TAC, and MDA after 12 weeks of intervention.

### Supplementary Information

**Additional file 1.**

## Data Availability

(ADM) The datasets used and/or analyzed during the current study are available from the corresponding author on reasonable request.

## Declarations

### Protocol registration number in Iranian Registry of Clinical Trials

IRCTID: IRCT20140907019082N9. Registration date: 2018-09-23.
